# Multidimensional Environmental Factors and Sleep Health for Aging Adults: A Focused Narrative Review

**DOI:** 10.3390/ijerph192315481

**Published:** 2022-11-22

**Authors:** Eunhwa Yang, Aliaa Ismail, Yujin Kim, Ece Erdogmus, Julie Boron, Felicia Goldstein, Jennifer DuBose, Craig Zimring

**Affiliations:** 1 School of Building Construction, Georgia Institute of Technology, Atlanta, GA 30332, USA; 2 Department of Gerontology, University of Nebraska Omaha, Omaha, NE 68182, USA; 3 Department of Neurology, School of Medicine, Emory University, Atlanta, GA 30322, USA; 4 SimTigrate Design Lab, Georgia Institute of Technology, Atlanta, GA 30332, USA

**Keywords:** built environment, psychosocial environments, sleep health, sleep disparities, aging adults

## Abstract

The timing, amount, and quality of sleep are critical for an individual’s health and quality of life. This paper provides a focused narrative review of the existing literature around multidimensional environments and sleep health for aging adults. Five electronic databases, Scopus, Web of Science, PubMed/Medline; EBSCOhost, PsycINFO (ProQuest), and Google Scholar yielded 54,502 total records. After removing duplicates, non-peer reviewed academic articles, and nonrelevant articles, 70 were included for review. We were able to categorize environmental factors into housing security, home environment, and neighborhood environment, and, within each environmental category, specific elements/aspects are discussed. This paper provides a comprehensive map connecting identified levels of influence (individual, home/house, and neighborhood-level) in which subfactors are listed under each level of influence/category with the related literature list. Our review highlights that multidimensional environmental factors can affect aging adults’ sleep health and eventually their physical, mental, and cognitive health and that sleep disparities exist in racial minorities in socioeconomically disadvantaged communities in which cumulative environmental stressors coexist. Based on this focused narrative review on the multidimensional sleep environments for aging adults, knowledge gaps are identified, and future research directions are suggested.

## 1. Introduction

The timing, amount, and quality of sleep are critical for an individual’s long-term health and quality of life. Inadequate sleep is linked to an increased risk of obesity [1,2,3,4] daytime fatigue [5], and a decline in cognitive performance [6,7]. Sleep deficiencies and disturbances can contribute to negative physical and mental health consequences, such as cardiovascular diseases [8], dementia [9], and a poorer quality of life [10]. Sleep patterns and architecture also change with age, causing prevalent sleep problems for aging adults [11,12]. In one study, nearly half of the aging participants reported chronic sleep-related complaints, and 10–20% reported difficulty initiating sleep [13]. The types of sleep disturbances for aging adults include insomnias, parasomnia, and disorders in sleep schedules, including waking too early and trouble falling asleep [14]. Other contributors of sleep disturbances include primary sleep disorders, circadian rhythm disturbances, medical and psychiatric conditions, medications, and dementia [15].

Some studies found that various environmental conditions can affect sleep health regardless of age, such as light [16], noise [16], indoor temperatures [17,18,19], and indoor air quality [19,20]. Suggested nighttime environments and behaviors for adults of any age include keeping the environment quiet, dark, and at a comfortable temperature, as well as having a similar bedtime routine with anyone who shares the room [21]. Most studies explored nighttime bedroom environments for the overall population without considering age differences. However, aging adults’ physiological and cognitive aging may require different environmental conditions for sleep. For example, aging can influence vision, especially with spatial contrast sensitivity, vision under low luminance, temporal sensitivity and motion perception, and visual processing speed [22]. Besides vision-related sensitivity, light exposure for circadian synchronization differs with aging, 75 year olds require triple the amount of lighting needed for a 45 year old to elicit the same circadian response due to changes in the eye [23]. Aging and hearing loss leads to increased neural responses to sounds and noise in aging adults compared to younger populations [24]. Aging is also associated with a progressive decrease in the perception of warmth versus cold [25].

Designing the appropriate type, location, and controls for a lighting source within reach and eliminating potential trip hazards along the route to the bathroom is important for all aging adults. Furniture, rugs, cords, flooring transitions or doorway thresholds, and other objects should be organized and designed to create a clear pathway for night-time movements. Over 50% of aging adults experience nighttime urination (also called nocturia) every night/almost every night due to physical changes in the urinary system, and another study reported that approximately 60% of older adults use the bathroom at least twice a night [26]. Within this group, nighttime urination is the leading cause of nocturnal sleep and sleep disturbance [27]. In addition to home environments, sleep quality can be affected by neighborhood environments such as traffic, pollution, and neighborhood density [16]. Exploring multilevel of environments will help the comprehensive understanding of the environmental effects on the sleep quality of aging adults.

This paper provides a focused narrative review on multilevel factors regarding the built environment that affect sleep health for aging adults from the perspectives of Bronfenbrenner’s ecological systems theory [28] and the National Institute on Minority Health and Health Disparities research framework [29]; we then propose a conceptual framework that can serve as a theoretical foundation for future studies to understand the key built environment mechanisms that contribute to sleep health.

## 2. Methods

A systematic literature review was conducted by searching four electronic databases, Scopus, Web of Science, PubMed/Medline; EBSCOhost, PsycINFO (ProQuest) for articles published up to January 2022 using the following search keywords: sleep health, aging adults, housing security, residence, house environment, and neighborhood environment (Table 1). A set of screening criteria was applied to the articles: (1) original research, (2) published in English in peer-reviewed journals, (3) content relevance including at least one component of any of the levels of the built environments and relationship between the built environment and sleep outcomes, and (4) providing empirical evidence. 

**Figure 1:**
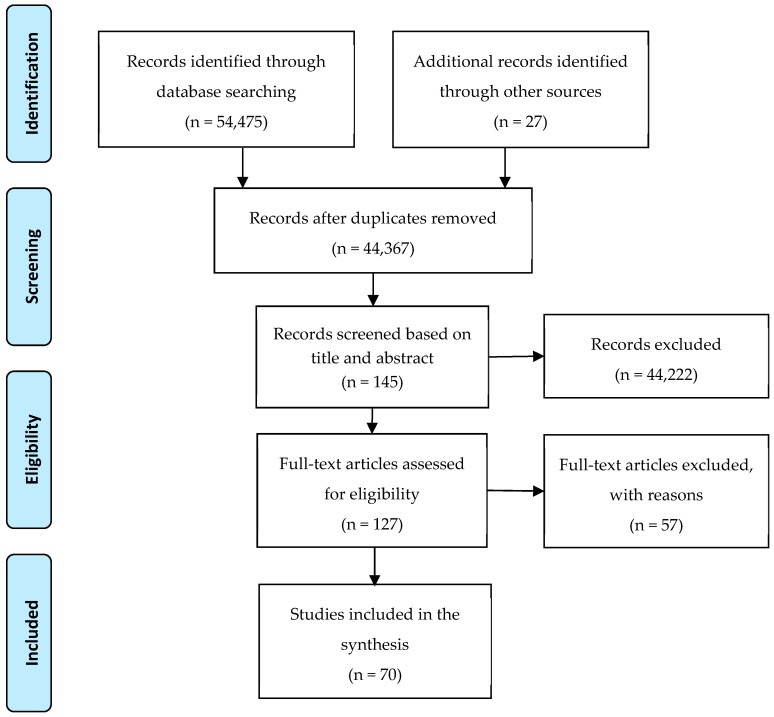
Literature search and screening process.

The literature search and screening process is shown in Figure 1. First, the search of the four databases yielded a total of 54,475 records. Additionally, 27 articles were identified through Google Scholar using the following combinations of keywords: built environment and sleep health, light and sleep health, noise and sleep health, crowding and sleep health, indoor temperature and sleep health, and neighborhood and sleep health. Once a collection of articles was formed, 10,135 duplicates were removed. The initial screening process also involved eliminating non-peer-review academic articles, including conference proceedings, abstracts only, letters, opinions, and dissertations. While reviewing the titles of the papers, those that were medical interventions and not targeting an aging population or related to the built environment were considered irrelevant. From this process, 44,074 papers were eliminated, resulting in 293 papers. After title screening, the authors read through the abstracts of 293 papers and eliminated 148 papers that did not include any empirical evidence or only discussed conceptual knowledge, resulting in 145 papers. Out of the 145 papers, the authors had full access to only 127 articles. After reading the full papers, 57 articles were excluded as they did not meet inclusion criteria (i.e., relation to an aging population, the built environment, empirical evidence). As a result, a total of 70 articles were included in the literature review. After that, these papers were categorized into three dimensions of built environments: (1) housing security, (2) home environment, and (3) neighborhood environment.

## 3. Results

### 3.1. Housing Security

Housing insecurity involves high housing costs in proportion to income, the frequency of moving, and the financial difficulty of paying rent. These issues naturally create significant psychological stress and cognitive burden, which in turn are expected to negatively impact the sleep health of aging adults with already diminishing cognitive health. A study with 4850 participants reported that non-homeowners and those without health insurance and food security were significantly more likely to report very short sleep (<5 h) [30]. Another study with 159,856 participants across 36 states in the United States linked low income with frequent sleep complaints [31]. Individuals who could not pay rent or mortgage payments due to economic resource deficits slept on average 22 fewer minutes at night and had lower sleep quality; additionally, participants who were evicted because of the inability to pay rent/mortgage payments slept 32 min fewer than those with stable housing conditions [32].

### 3.2. House/Household Environment

At the level of a home environment, we found four major factors that affect sleep health in the ambient environment: (1) lighting, (2) thermal comfort, (3) noise and (4) crowding.

First, light plays a vital role in regulating the human’s circadian system, the body’s biological clock that controls when to be active and when to rest. Poor light exposure can disrupt the 24 h sleep cycle [33]. A study reported that the amount of 24 h illumination aging adults are exposed to is correlated with shorter sleep latencies (r = 0.29, *p* < 0.001, two-tailed) and less expression of depressed moods (r = 0.21, *p* = 0.01, two-tailed) [34]). Lighting interventions sometimes become necessary when aging adults are not exposed to enough daylight throughout the day. The optimal lighting arrangement for circadian rhythm entrainment is a high level of blue-enriched white light in the morning to phase-advance the circadian clock (move the rhythm earlier in the day), and medium to high levels of neutral white light during mid-day to increase alertness without phase-shifting effects on aging adults’ circadian rhythm and, in the evening, dimmed illumination (yellowish-white light) to avoid phase-delay to the circadian rhythm (move the rhythm later in the day) [35]. Aging adults with Alzheimer’s disease when exposed to light sessions of approximately 2500 full spectrum light had significantly (*p* < 0.05) less total wake time and better sleep efficiency after the exposure [36]. Artificial light at night inhibits the secretion of melatonin, a hormone involved in sleep regulation [37,38]. A study that measured the indoor illumination level at night for 857 aging adults reported that the highest quartile of light intensity (mean 9.7 lux) showed higher odds for insomnia (OR 1.61, 95% CI 1.05–2.45) and negatively affected subjective and objective sleep quality [39]. Another study associated night-time artificial lighting levels of 5 lux or greater with an increased risk of depression [40]. Artificial light at night also delayed bedtime [41], increased subsequent sleep-onset latency by 17 min [42], and lengthened wake time after sleep onset [41]. It was also found that frail aging adults who spend most of their time indoors in bed do not get exposed to enough illumination throughout the day. A study with 44 participants in Japan measured the subjects’ illumination levels throughout the day for 3 days found that the mean illuminance while indoors was just 201 lux and for bed ridden adults was only 5 ± 11 lux [43]. 

Second, ambient temperature is also a key factor influencing sleep quality in aging populations [19]. Low indoor temperatures have been linked to extended sleep onset latency [13]. High temperatures have also been found to influence sleep health. A study conducted in the summer in Gunma, Japan measured the bedroom temperature for the participants to be between 25 °C and 28 °C (77 to 82.4 °F), and reported that at this temperature range, aging adults’ sleep was significantly more disturbed than that of younger adults [44]. For older males in Japan, an experiment examined two sets of conditions for sleep: 26 °C (78.8 °F) with 50% relative humidity (RH) and 32 °C (89.6 °F) with 50% RH. The study found that the higher temperature of 32 °C (89.6 °F) increased wakefulness and suppressed REM sleep [18]. Another study targeting older men in Japan associated a temperature of 28 °C (82.4 °F) with increased wake time after sleep onset and impaired sleep quality [41]. During summer, when air temperature increased by 1 °C (1.8 °F), sleep efficiency decreased by 0.72%, and REM sleep duration decreased by 2.10 min for older adults in China [19]. While these findings suggest relatively modest changes to sleep due to temperature, it is interesting to note that the older adults experience greater impact on their sleep due to elevated temperatures compared to a similar study of young people [45,46]. However, engineers and designers broadly use the American Society of Heating, Refrigerating, and Air-Conditioning Engineers (ASHRAE) Standard 55 and ISO 7730:2005 as thermal comfort standards, which are based on studies of younger adults and assume that similar conditions also apply to aging adults. When given control over their environment, aging adults are noted to be less precise in adjusting their thermal environment; they are reported to have reduced sensory skills to identify the immediate need for more warmth or coolness compared to younger adults. It is assumed that the inability to discriminate temperature differences is related to both cognitive and physical deterioration [47].

Third, noise is also reported as one of the factors that negatively affects sleep quality for older adults [48]. A study with 231 older adults tested the association of household environment and in-bed behaviors with sleep duration and sleep efficiency; the study reported a standard deviation increase in an adverse household environment was associated a low self-reported sleep duration (β = −13.9 min, 95% confidence interval: −26.1, −1.7) and actigraphy-based sleep efficiency (β = −0.7%, −1.4, 0.0) [49]. It should be noted that the adverse house environment in [49] study included safety, physical comfort, temperature, noise, and light disturbances, and the all of these environments showed an association with sleep as a combined effect. Awakening or arousals during sleep occur for most individuals when the noise level is between 40 to 80 dB. However, this range can also be influenced by individual characteristics such as aging and noise sensitivity [50]. According to the World Health Organization (WHO) guidelines for nighttime noise, published in 2009, the annual average noise exposure at night should not exceed 40 dB. Exposure to sounds exceeding 40 dB could lead to insomnia, sleep disturbance, and other health consequences such as elevated blood pressure and heart attacks [51]. Household noise was one of the perceived factors associated with self-reported short sleep duration for aging adults [49]. Another study reported that in older adults with insomnia, their bedrooms were not completely quiet at night and the researchers suspected that aging adults ignore or downplay the consequences of nocturnal noise [52].

Lastly, large households and crowding may also contribute to patterns of insufficient sleep [53]. The federal government defines household crowding based on the number of persons per room [54]. According to the American Crowding Index (ACI), crowding occurs if there is more than one person per room; severe crowding occurs if more than three people occupy two rooms—where rooms exclude bathrooms, half-rooms, foyers, and porches. A study with 371 adults living in urban low-income housing in the United States found that household crowding, where crowding was defined according to the ACI (the ratio of persons to rooms ≥ 1), was associated with lower likelihood of getting long sleep (≥8.5 h), relative to both average (6.5–8.5 h) and short sleep duration (≤6.5 h) [55].

At the other end of the spectrum of crowding, older adults are at a higher risk of experiencing negative health consequences from social isolation and loneliness [56], especially aging adults living alone. Studies have linked social isolation and loneliness to poor sleep health for aging adults. A nationally representative U.S. sample of 759 older adults showed social isolation was associated with more time in bed and worse objective sleep quality [57]. Social isolation was associated with lower self-reported sleep quality [58]. Loneliness is also associated with shorter self-reported sleep and insomnia symptoms [57].

### 3.3. Neigborhood Environment

Factors such as noise, lighting, outdoor air pollution, perceived safety, and walkability are associated with neighborhood environments [16]. There has been an effort to provide noise-free public environments to improve public health and the Noise Control Act was established in 1972 by the U.S. Environmental Protection Agency (EPA). Neighborhood noise is generated from various sources, including road traffic, railways, and aircraft. According to one study, outdoor noise was measured between 45 dB and 65 dB during the night [59]. According to a laboratory experiment, the possibility of sleep disturbance in older males (aged 55–75 years) increased as the peak traffic noise increased from 40 dB to 80 dB [60]. In one study, loud (>50 dB) traffic noise events heard from the home environment increased the possibility of ectopic heartbeats in aging adults [61]. Along with the measured noise in dB, noise can also be measured indirectly. For example, one study correlated noise to the distance between home and main roads. For a sample of people aged 50 and older, the distance from home to main roads within 50 m (54.7 yards) showed a stronger association with a very low-frequency index, which causes sleep fragmentation, compared to the group residing in homes that are a distance within 100 m (109.4 yards) [62]. Notably, aging adults (aged 55–75 years) were less sensitive to awakening than the middle-aged (46–51 years) group as hearing impairment is considered one of the features of aging adults [60].

One study examined the relationships between neighborhood artificial lights at night and the use of hypnotic medications (including zolpidem and triazolam) by older adults aged over 60 years [63]. This population-based study used 52,027 survey responses, and the neighborhood artificial lighting was measured and provided by the National Centers for Environmental Information in South Korea. For the outdoor lighting, the visible light intensity data ranged between 0 to 63 nanowatts/cm^2^/sr, and they were categorized into quartiles and it was found that the increase in the artificial outdoor lighting exposure was associated with an increase in the length of prescription days and the daily dose of hypnotic medications.

Exposure to outdoor air pollution can negatively impact the immune system of older people and be negatively associated with sleep quality. In a city, traffic- or industry-related chemicals, including nitrogen dioxide (NO_2_), sulfur dioxide (SO_2_), ozone (O_3_), ambient fine particulate matter (PM2.5), and inhalable particles (PM10), cause massive air pollution. [64] examined the relationship between the short-term exposures to air pollution and sleep disorders for individuals between 60 and 106 years (mean age 76 years) in a city in China. Aging adults between 60 and 75 years old showed higher risks of sleep disorder with the higher PM2.5 (min = 4.24, med = 37.69, and max = 440 µg m^−3^), PM10 (min = 10.18, med = 60.11, and max = 545 µg m^−3^), and NO_2_ (min = 5.59, med = 37.96, and max = 118 µg m^−3^); on the other hand, those 75 years and older showed a stronger relationship between O_3_ (min = 0, med = 62.58, and max = 398 µg m^−3^) and sleep disorder [64].

Neighborhood socio-environments can be measured in physical disorder and social cohesion. A study, with 7231 community-dwelling participants, measured the perceived neighborhood physical disorder (vandalism, rubbish, vacant houses, and perceived safety walking alone at night) and social cohesion (feeling part of the area, trusting people, friendliness of people, and availability of help if in trouble) of people aged 50 and older using a seven-point Likert scale (1 = favorable, 7 = worse) and analyzed them with respect to the sleep disorder [65]. The increase in the perceived neighborhood physical disorder and the poor social cohesion were positively correlated with trouble falling asleep. Similarly, according to the U.S. National Health Interview Survey (NHIS) from 2013 and 2018 [66], adults aged 50 and older in low social-cohesion neighborhoods were more likely to report very short sleep (less than six hours), trouble falling asleep, trouble staying asleep, and insomnia symptoms than those living in socially cohesive neighborhoods. Specifically, African Americans (AAs) and Hispanic aging adults reported low sleep health in terms of insomnia symptoms, and older Hispanic women showed significantly very short sleep in less socially cohesive neighborhoods compared to other ethnic, gender, and age groups [66]. It was also found that the positive relationship between social cohesion and sleep duration of aging adults was significant among AAs [67].

### 3.4. Institutional Settings: Nursing Homes and Hospitals

The multi levels of influence—housing security, house/household environment, and neighborhood environment—mentioned above are focused on the analysis of individuals’ financial, housing, and neighborhood-level conditions. Beside the individual home settings, 5% of aging adults reside in institutional settings, and this percentage increases to 20% for individuals over age 80 [68]. At the level of Institutional settings, two common living arrangements were identified for the elderly, nursing homes and hospital environments. For nursing homes, a study of 540 participants residing in three different nursing homes in Turkey found that the sleep quality for over half of the subjects (60.9%) was poor [69]. Another study in Iran studied 711 aging adults with insomnia, residing in nursing homes, reported that the sleep environment satisfaction had a significant correlation with insomnia (*p* = 0.001) [70].

Through the literature review, we found that three factors had the most significant impact on aging adults’ sleep health in nursing homes: thermal comfort, indoor illumination, and noise. First, the indoor temperature exceeded 28 °C during the night in the summer because of the air conditioners being turned off while the caregivers were absent from the nursing home, which affected the residents’ sleep quality in Japan [71]. In that same study, it was also reported that adaptive approaches of turning on a fan or opening a window were not enough to improve the sleep efficiency, especially for older men with thermoregulation deterioration [71].

Second, nursing home residents are often exposed to less daylight during the day, which is necessary for good sleep health. A study in Spain investigated the relationship between access to daylight and circadian rhythm in two nursing homes and reported that the residents of the nursing home with more daylight and wider windows had a healthier circadian rhythm, better sleep quality, and improved autonomic function than those living in rooms with less daylight and limited window spaces [72]. The mean daylight exposure for 66 aging adults was 485 lux (SD = 761), 17% of aging adults living in a nursing home were never exposed to light (combined daylight and artificial light) greater than 1000 lux and 26% of patients were not exposed to any light greater than 2000 lux [73]. Lighting interventions are found to be a promising treatment to mitigate circadian rhythm disturbance in aging adults; when aging adults with insomnia were exposed to an intensity of approximately 2500 lux of bright light at eye level for 4 h during midday, nocturnal melatonin secretion increased without circadian phase-shifting [33]. In an experiment, 66 participants in a nursing home were exposed to approximately 2500 lux, similar to the intensity of a cloudy day, through a low glare lighting system and reported that nighttime sleep of participants with severe or very severe dementia increased by 16 min (*p* = 0.008) when exposed in the morning and by 14 min (*p* = 0.01) when exposed to 2500 lux for 8.4 h a day [74].

Lastly, noise is the final environmental attribute that was found to affect nursing home residents’ sleep health. Noise at night was associated with poor sleep quality (β = −0.20, *p* < 0.001) when the sleep of 125 nursing home residents was objectively and subjectively measured [75]. A study with 48 participants reported that 48% of the subjects were disturbed by noise, either made by other residents or the nurses or both, yet when the researchers suggested using earplugs, only 3 out of 17 participants were interested in trying them [76]. Another study also identified noise as a key issue causing sleep disturbances through semi-structured interviews with 38 aging adults residing in 4 different nursing homes [77]. These interview findings corroborate objective measure of noise and sleep interruption; environmental noise and light recorded with two-minute intervals for 118 aging adults indicated that noise was associated with 50% of all waking episodes of four minutes of longer [78].

In the same study with 118 aging adults, the average of 32 noise occurrences per night for each participant were recorded. The sources of noise was either nurses entering the room for continence-related checks or providing care for residents such as administering medications [78]. As a follow up intervention study, educational and behavioral interventions were introduced to staff and nurses from eight different nursing homes, and although the noise was reduced it was not enough to improve aging adults’ sleep quality [79].

In hospital environments, the effect of lighting on older patients’ sleep was examined. A study conducted in the Netherlands found that providing a dynamic lighting cycle for 20 days improved the objective and subjective sleep quality of cardiology patients compared to patients with standard lighting [80]. In terms of lighting colors, there was no significant difference in sleep duration of patients with dementia between lighting with white and red colors according to quantitative data, but the staff interviews found that the patients slept better and were less agitated at night under the red lighting conditions [81]. Notably, as other environmental elements were not tested, the findings may be affected by the patient types of the studies.

## 4. Discussion

**Figure 2:**
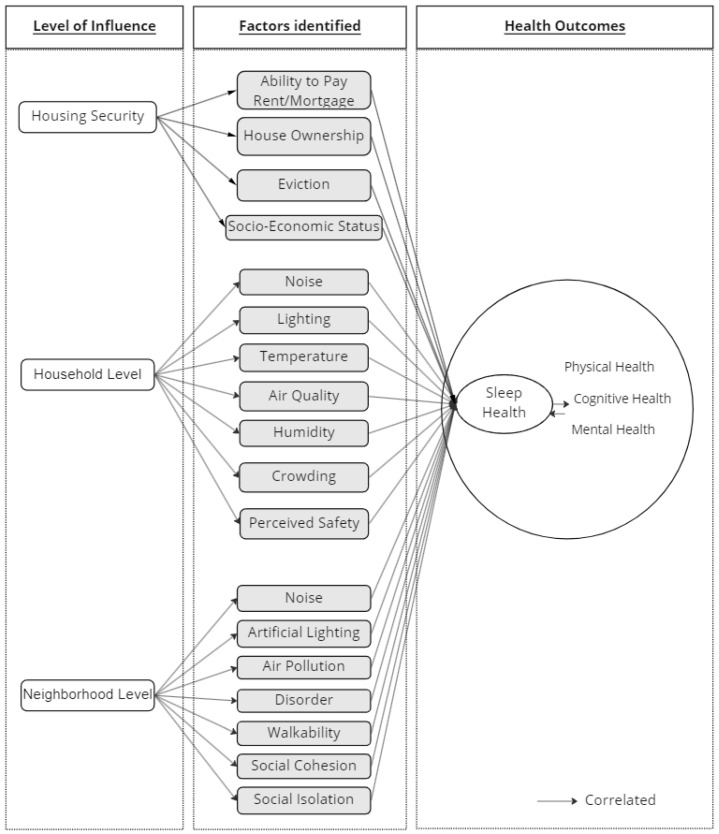
Ecological systems of multilevel factors influencing individuals’ sleep health.

Understanding sleep and the contributing factors and underlying mechanisms that affect sleep is a complex task. This review paper specifically focuses on findings from the existing literature regarding the ecology of sleep and multilevel factors that affect aging adults’ sleep health from the environmental perspective. Based on the 70 articles reviewed for this study, a map of sleep ecology with respect to built environments (Figure 2) is proposed in this paper. The proposed environmental sleep ecology map was developed based on the perspectives of Bronfenbrenner’s ecological systems theory [28] and the National Institute on Minority Health and Health Disparities research framework [29]. Ref. [82] also proposed a social ecological model of sleep based on Bronfenbrenner’s ecological systems theory. Ref. [82]’s model embraces many factors within each level (i.e., individual level: genetics, health, beliefs, attitudes, choices, etc.; social level: home, work, neighborhood, race/ethnicity, SES, religion, culture, etc.; societal level: technology, public policy, globalization, environment, geography, etc.); whereas, our proposed sleep ecology map foregrounds environmental factors at each level.

First, housing insecurity has a strong association with individuals’ sleep deficiencies, complaints, and quality. Sleep complaints were found to be associated with socioeconomic status; lower SES groups reported higher sleep complaints [31]. However, in the current literature, the exploration of sleep disparities due to housing insecurity for older adults with low SES is limited. Thus, there is a great need to determine the ecology of sleep health for aging racial minorities with low SES. Although there has been a long movement in understanding and defining “housing insecurity,” such as the United Nations (U.N) General Assembly in Article 25 of the 1948 Universal Declaration of Human Rights [83], the U.S. Housing Act of 1949 [84], and the U.S. Department of Health and Human Services in 1998 [85], there is also a great need to develop a uniform/standardized measurement tool to assess housing insecurity [86]. In 2019, the American Housing Survey (AHS), sponsored by the Department of Housing and Urban Development (HUD) and conducted by the U.S. Census Bureau, implemented the housing insecurity research module and conducted 15 cognitive interviews, which tested a questionnaire on housing insecurity which consisted of 15 questions on “screener” (i.e., home type, tenure), 25 questions on “affordability” (i.e., difficulty affording housing), 35 questions on “stable occupancy” (i.e., frequency of moving, forced move, temporary housing conditions), and 10 questions on “decent and safe” (i.e., home safety and security, deferred maintenance) [87]. The AHS housing insecurity questionnaire is an effort to develop a housing insecurity index to bring more consistency in defining and measuring housing insecurity for researchers and policymakers. The cognitive interviews of the AHS housing insecurity questionnaire had a limited sample size and across ages (not specifically for aging adults); however, given that the full survey items and detailed interview protocol reported and shared to the public via the U.S. Census Bureau have the potential to be permanently implemented toward the AHS that is conducted every two years, this has the potential to be very beneficial for scholars and policymakers. In addition to the AHS housing insecurity questionnaire, there can be another layer stemming from ownership of homes specifically for aging adults. Typically, owning or renting a home affects how the minor and major repairs are handled, managed, and paid. The AHS housing insecurity questionnaire addressed the owners’ responsiveness for repairs/fixes when the respondents were tenants; yet the homeowners’ capabilities to address repairs and fixes (i.e., find, manage, and pay for technicians and contractors) were not assessed when the respondents were the owners of the homes. Aging adults who own and reside in their homes may have difficulty finding the right technicians, contractors, and financial resources to repair and fix problems in the house that may compromise the ambient environment at home.

Second, the ambient home environment, such as lighting, thermal comfort, noise, and crowding, has been found to include important factors for sleep health. A minimum intensity of 2500 lux of light (daylight or electric) during the daytime is beneficial for sleep health; yet studies found that the lack of light exposure, especially in institutional settings (i.e., nursing homes). Additionally, aging adults tend to be less sensitive about discriminating temperature differences due to cognitive and physical deterioration compared to young adults, yet the literature provides consistent evidence of the negative impact of elevated temperature (i.e., >77 °F) on sleep disturbances. However, current industry practices are based on the ASHRAE Standard and guidelines that are determined from studies of younger adults, assuming the same individual perception and thermal comfort for aging adults. There was a consistent finding that noise levels exceeding 40 dB increase sleep disturbances and reduce sleep duration. In terms of crowding, ACI does not account for other factors that may affect crowding, such as age, gender of the household members, and their relationship. This Index may also not be culturally appropriate for ethnic minority groups, as these groups may have a different understanding of what is considered a crowded household. At the other end of the spectrum of crowding, there are two aspects of social isolation that can affect aging adults’ sleep health: social disconnectedness, which is described as the physical separation from others, and perceived isolation, which is the feeling of loneliness as well as the perceived lack of social support [88]. Studies have linked social isolation and loneliness to sleep health (poorer sleep quality and short sleep duration) for aging adults.

Third, neighborhood-level factors, such as noise, lighting, outdoor air pollution, perceived safety, and walkability, were studied in different regions around the world. Noises louder than 40 dB and homes close to the main road (within 54.7 yards) show stronger associations with sleep fragmentation and poorer sleep health. Higher outdoor lighting levels and air pollution were also negatively associated with sleep quality for aging adults. Neighborhood physical disorder and social cohesion were correlated with sleep health; interestingly, there seems to be an interaction between neighborhood social cohesion by race on sleep duration with AAs and Hispanics showing a stronger relationship between neighborhood social cohesion and their sleep health than other races.

Lastly, although this narrative review is more focused on individual housing security, home/household environment, and neighborhood environment in individuals’ home settings, the authors also found interesting studies on environmental conditions and sleep health for aging adults in institutionalized settings, such as nursing homes and hospitals. Three main factors are discussed in this context: thermal comfort, indoor lighting level, and noise. These results corroborate the findings from the individual home setting studies regarding the built environment-related causes of sleep disturbances; however, the sources of these disturbances, the level of adjustments the individuals can make on the building system controls, as well as the mechanical systems (e.g., heating, ventilation, and air conditioning (HVAC)), lighting, and acoustic conditions/installations are significantly different from individual home settings.

## 5. Conclusions

In summary, there is a general consistency of environmental factors that affect the sleep health of aging adults. However, there are two important gaps in the current literature: (1) studies that investigate to what extent cumulative multilevel environmental factors affect sleep health when individuals with low SES are simultaneously exposed to less desirable housing security, home (physical and social environments), neighborhood, community-level environments; (2) studies that identify the most critical factor among multidimensional built environmental factors, which can provide evidence-based housing design/construction/management and policy development. Some of the existing literature specifically analyzed sleep health in low-income communities and racial minorities; however, the majority of the studies that have been identified in this review do not specifically test the association or interaction effect of race and SES in aging adults. Further, historically, AAs and Hispanics have been underrepresented in sleep and environmental studies. Thus, there is great value for future research to investigate these multilevel environments and their cumulative exposure, which often coexists in underserved communities.

Several studies suggested interventions for the built environment to improve sleep health, but there is a lack of studies that do so for the intersectionality of the demographics: race/ethnic minorities, low SES, and aging/cognitive decline. Interventions should be culturally appropriate, inexpensive to obtain and maintain, and easy to adopt for these demographics. There is also a great need to develop design guidelines for homes with aging in mind, in which residential design, construction, and management practices can refer to in order to help individuals who wish to stay home throughout their lifetime and as long as possible, beyond providing supportive environments for better sleep health.

## Figures and Tables

**Table 1:** Search keywords algorithms.

Sleep		(1) sleep (2) insomnia (3) sleep disparities (4) sleep health (5) abnormal sleep (6) sleep disorder (7) sleep-wake (8) sleep quality (9) sleep apnea (10) sleep efficiency (11) sleep architecture (12) sleep duration (13) sleep difficulty
Individual		(1) older adult (2) aging adult (3) elderly
Built environment	Housing security	(1) rent payment OR mortgage payment (2) housing affordability (3) homelessness
	Home environment	(1) light (2) noise (3) temperature OR cold (4) ventilation (5) air (6) indoor green space OR indoor gardening (7) window
	Neighborhood	(1) crime (2) litter OR cleanliness (3) pleasantness (4) safety OR violence (5) social cohesion (6) social capital (7) social disadvantage (8) neighborhood socioeconomic (9) walkability OR walking environment (10) crowding (11) green space OR tree OR park OR forest (12) civic participation (13) reciprocity (14) traffic (15) availability of healthy food
Other	Institutional setting	(1) nursing home (2) hospitalization

## Data Availability

Not applicable.

## References

[1] Beccuti G., Pannain S. (2011). Sleep and obesity. Curr. Opin. Clin. Nutr. Metab. Care.

[2] Buxton O.M., Marcelli E. (2010). Short and long sleep are positively associated with obesity, diabetes, hypertension, and cardiovascular disease among adults in the United States. Soc. Sci. Med..

[3] Ogilvie R.P., Patel S. (2018). The epidemiology of sleep and obesity. Sleep Health.

[4] Reither E.N., Krueger P.M., Hale L., Reiter E.M., E Peppard P. (2014). Ethnic variation in the association between sleep and body mass among US adolescents. Int. J. Obes..

[5] Elmenhorst E.-M., Elmenhorst D., Luks N., Maass H., Vejvoda M., Samel A. (2008). Partial sleep deprivation: Impact on the architecture and quality of sleep. Sleep Med..

[6] Killgore W.D. (2010). Effects of sleep deprivation on cognition. Prog. Brain Res..

[7] Willette-Murphy K., Todero C., Yeaworth R. (2006). Mental health and sleep of older wife caregivers for spouses with alzheimer’s disease and related disorders. Issues Ment. Health Nurs..

[8] Suzuki J.-I., Isobe M., Morishita R., Nagai R. (2009). Tea Polyphenols Regulate Key Mediators on Inflammatory Cardiovascular Diseases. Mediat. Inflamm..

[9] Shi L., Chen S.-J., Ma M.-Y., Bao Y.-P., Han Y., Wang Y.-M., Shi J., Vitiello M.V., Lu L. (2017). Sleep disturbances increase the risk of dementia: A systematic review and meta-analysis. Sleep Med. Rev..

[10] Lee M., Choh A.C., Demerath E.W., Knutson K., Duren D.L., Sherwood R., Sun S.S., Chumlea W.C., Towne B., Siervogel R.M. (2009). Sleep disturbance in relation to health-related quality of life in adults: The fels longitudinal study. J. Nutr. Health Aging.

[11] Muehlroth B.E., Werkle-Bergner M. (2020). Understanding the interplay of sleep and aging: Methodological challenges. Psychophy.

[12] Ohayon M.M., Carskadon M.A., Guilleminault C., Vitiello M.V. (2004). Meta-Analysis of Quantitative Sleep Parameters From Childhood to Old Age in Healthy Individuals: Developing Normative Sleep Values Across the Human Lifespan. Sleep.

[13] Saeki K., Obayashi K., Tone N., Kurumatani N. (2015). A warmer indoor environment in the evening and shorter sleep onset latency in winter: The HEIJO-KYO study. Physiol. Behav..

[14] Ancoli-Israel S. (2005). Sleep and aging: Prevalence of disturbed sleep and treatment considerations in older adults. J. Clin. Psychiatry.

[15] Martin J.L., Ancoli-Israel S. (2008). Sleep Disturbances in Long-Term Care. Clin. Geriatr. Med..

[16] A Johnson D., A Hirsch J., A Moore K., Redline S., Roux A.V.D. (2018). Associations Between the Built Environment and Objective Measures of Sleep. Am. J. Epidemiol..

[17] Okamoto-Mizuno K., Mizuno K., Michie S., Maeda A., Lizuka S. (1999). Effects of Humid Heat Exposure on Human Sleep Stages and Body Temperature. Sleep.

[18] Okamoto-Mizuno K., Tsuzuki K., Mizuno K. (2004). Effects of mild heat exposure on sleep stages and body temperature in older men. Int. J. Biometeorol..

[19] Yan Y., Lan L., Zhang H., Sun Y., Fan X., Wyon D.P., Wargocki P. (2022). Association of bedroom environment with the sleep quality of elderly subjects in summer: A field measurement in Shanghai, China. Build. Environ..

[20] Lan L., Sun Y., Wyon D.P., Wargocki P. (2021). Pilot study of the effects of ventilation and ventilation noise on sleep quality in the young and elderly. Indoor Air.

[21] Neikrug A.B., Ancoli-Israel S. (2009). Sleep Disorders in the Older Adult—A Mini-Review. Gerontology.

[22] Owsley C. (2011). Aging and vision. Vis. Res..

[23] Turner P.L., Mainster M.A. (2008). Circadian photoreception: Ageing and the eye’s important role in systemic health. Br. J. Ophthalmol..

[24] Herrmann B., Maess B., Johnsrude I.S. (2018). Aging Affects Adaptation to Sound-Level Statistics in Human Auditory Cortex. J. Neurosci..

[25] Guergova S., Dufour A. (2011). Thermal sensitivity in the elderly: A review. Ageing Res. Rev..

[26] Bosch J.R., Weiss J.P. (2010). The Prevalence and Causes of Nocturia. J. Urol..

[27] Bliwise D.L., Foley D.J., Vitiello M.V., Pour Ansari F., Ancoli-Israel S., Walsh J.K. (2009). Nocturia and disturbed sleep in the elderly. Sleep Med..

[28] Bronfenbrenner U. (1979). The Ecology of Human Development: Experiments by Nature and Design.

[29] Alvidrez J., Castille D., Laude-Sharp M., Rosario A., Tabor D. (2019). The National Institute on Minority Health and Health Disparities Research Framework. Am. J. Public Health.

[30] Whinnery J., Jackson N., Rattanaumpawan P., Grandner M.A. (2014). Short and Long Sleep Duration Associated with Race/Ethnicity, Sociodemographics, and Socioeconomic Position. Sleep.

[31] Grandner M.A., Patel N.P., Gehrman P.R., Xie D., Sha D., Weaver T., Gooneratne N. (2010). Who gets the best sleep? Ethnic and socioeconomic factors related to sleep complaints. Sleep Med..

[32] Bozick R., Troxel W.M., A Karoly L. (2021). Housing insecurity and sleep among welfare recipients in California. Sleep.

[33] Mishima K., Okawa M., Shimizu T., Hishikawa Y. (2001). Diminished Melatonin Secretion in the Elderly Caused by Insufficient Environmental Illumination1. J. Clin. Endocrinol. Metab..

[34] Wallace-Guy G.M., Kripke D.F., Jean-Louis G., Langer R.D., Elliott J.A., Tuunainen A. (2002). Evening light exposure: Implications for sleep and depression. J. Am. Geriatr. Soc..

[35] Shishegar N., Boubekri M., Stine-Morrow E.A., Rogers W.A. (2021). Tuning environmental lighting improves objective and subjective sleep quality in older adults. Build. Environ..

[36] McCurry S.M., Pike K.C., Vitiello M.V., Logsdon R.G., Larson E.B., Teri L. (2011). Increasing Walking and Bright Light Exposure to Improve Sleep in Community-Dwelling Persons with Alzheimer’s Disease: Results of a Randomized, Controlled Trial. J. Am. Geriatr. Soc..

[37] Cajochen C. (2007). Alerting effects of light. Sleep Med. Rev..

[38] Lewy A.J., Wehr T.A., Goodwin F.K., Newsome D.A., Markey S.P. (1980). Light Suppresses Melatonin Secretion in Humans. Science.

[39] Obayashi K., Saeki K., Kurumatani N. (2014). Association between light exposure at night and insomnia in the general elderly population: The HEIJO-KYO cohort. Chronobiol. Int..

[40] Obayashi K., Saeki K., Kurumatani N. (2017). Bedroom Light Exposure at Night and the Incidence of Depressive Symptoms: A Longitudinal Study of the HEIJO-KYO Cohort. Am. J. Epidemiol..

[41] Tsuzuki K., Mori I., Sakoi T., Kurokawa Y. (2015). Effects of seasonal illumination and thermal environments on sleep in elderly men. Build. Environ..

[42] Obayashi K., Saeki K., Iwamoto J., Okamoto N., Tomioka K., Nezu S., Ikada Y., Kurumatani N. (2013). Effect of exposure to evening light on sleep initiation in the elderly: A longitudinal analysis for repeated measurements in home settings. Chrono- Int..

[43] Ichimori A., Tsukasaki K., Koyama E. (2015). Illuminance, Subjective Sleep Quality, and Psychosomatic Health in Elderly Individuals Requiring Care: A Survey of Japan’s Hokuriku Region in Winter. J. Community Health Nurs..

[44] Ohnaka T., Tochihara Y., Kanda K. (1995). Body Movements of the Elderly during Sleep and Thermal Conditions in Bedrooms in Summer. Appl. Hum. Sci..

[45] Hashiguchi N., Tochihara Y., Ohnaka T., Tsuchida C., Otsuki T. (2004). Physiological and subjective responses in the elderly when using floor heating and air conditioning systems. J. Physiol. Anthr. Appl. Hum. Sci..

[46] Natsume K., Ogawa T., Sugenoya J., Ohnishi N., Imai K. (1992). Preferred ambient temperature for old and young men in summer and winter. Int. J. Biometeorol..

[47] Collins K.J., Exton-Smith A.N., Dore C. (1981). Urban hypothermia: Preferred temperature and thermal perception in old age. BMJ.

[48] Thichumpa W., Howteerakul N., Suwannapong N., Tantrakul V. (2018). Sleep quality and associated factors among the elderly living in rural Chiang Rai, northern Thailand. Epidemiol. Health.

[49] A Johnson D., Jackson C.L., Guo N., Sofer T., Laden F., Redline S. (2021). Perceived home sleep environment: Associations of household-level factors and in-bed behaviors with actigraphy-based sleep duration and continuity in the Jackson Heart Sleep Study. Sleep.

[50] Basner M., McGuire S. (2018). WHO Environmental Noise Guidelines for the European Region: A Systematic Review on Environmental Noise and Effects on Sleep. Int. J. Environ. Res. Public Health.

[51] World Health Organization (2009). Regional Office for Europe. Night Noise Guidelines for Europe.

[52] Desaulniers J., Desjardins S., Lapierre S., Desgagné A. (2018). Sleep Environment and Insomnia in Elderly Persons Living at Home. J. Aging Res..

[53] Grandner M.A., Jackson N.J., Izci-Balserak B., Gallagher R.A., Murray-Bachmann R., Williams N.J., Patel N.P., Jean-Louis G. (2015). Social and Behavioral Determinants of Perceived Insufficient Sleep. Front. Neurol..

[54] Blake K.S., Kellerson R.L., Simic A. (2007). Measuring Overcrowding in Housing.

[55] Chambers E.C., Pichardo M.S., Rosenbaum E. (2016). Sleep and the Housing and Neighborhood Environment of Urban Latino Adults Living in Low-Income Housing: The AHOME Study. Behav. Sleep Med..

[56] Shankar A., McMunn A., Demakakos P., Hamer M., Steptoe A. (2017). Social isolation and loneliness: Prospective associations with functional status in older adults. Health Psychol..

[57] A Benson J., McSorley V.E., Hawkley L.C., Lauderdale D.S. (2021). Associations of loneliness and social isolation with actigraph and self-reported sleep quality in a national sample of older adults. Sleep.

[58] Yu B., Steptoe A., Niu K., Ku P.-W., Chen L.-J. (2018). Prospective associations of social isolation and loneliness with poor sleep quality in older adults. Qual. Life Res..

[59] Miedema H.M.E., Vos H. (1998). Exposure-response relationships for transportation noise. J. Acoust. Soc. Am..

[60] Thiessen G.J. (1978). Disturbance of sleep by noise. J. Acoust. Soc. Am..

[61] Carter N., Ingham P., Tran K., Hunyor S. (1994). A Field Study of the Effects of Traffic Noise on Heart Rate and Cardiac Arrhythmia During Sleep. J. Sound Vib..

[62] Gerbase M., Dratva J., Germond M., Tschopp J., Pépin J., Carballo D., Künzli N., Probst-Hensch N., Adam M., Stutz E.Z. (2014). Sleep fragmentation and sleep-disordered breathing in individuals living close to main roads: Results from a population-based study. Sleep Med..

[63] Min J.-Y., Min K.-B. (2018). Outdoor Artificial Nighttime Light and Use of Hypnotic Medications in Older Adults: A Population-Based Cohort Study. J. Clin. Sleep Med..

[64] Tang M., Li D., Liew Z., Wei F., Wang J., Jin M., Chen K., Ritz B. (2020). The association of short-term effects of air pollution and sleep disorders among elderly residents in China. Sci. Total Environ..

[65] Chen-Edinboro L.P., Kaufmann C.N., Augustinavicius J.L., Mojtabai R., Parisi J.M., Wennberg A.M.V., Smith M.T., Spira A.P. (2014). Neighborhood physical disorder, social cohesion, and insomnia: Results from participants over age 50 in the Health and Retirement Study. Int. Psychogeriatr..

[66] Alhasan D.M., Gaston S.A., Jackson W.B., Williams P.C., Kawachi I., Jackson C.L. (2020). Neighborhood Social Cohesion and Sleep Health by Age, Sex/Gender, and Race/Ethnicity in the United States. Int. J. Environ. Res. Public Health.

[67] Johnson D.A., Simonelli G., Moore K., Billings M., Mujahid M.S., Rueschman M., Kawachi I., Redline S., Roux A.V.D., Patel S.R. (2017). The Neighborhood Social Environment and Objective Measures of Sleep in the Multi-Ethnic Study of Atherosclerosis. Sleep.

[68] Mynatt E.D., Essa I., Rogers W. Increasing the opportunities for aging in place. Proceedings of the 2000 Conference on Universal Usability.

[69] Eser I., Khorshid L., Çinar S. (2007). Sleep Quality of Older Adults in Nursing Homes in Turkey: Enhancing the Quality of Sleep Improves Quality of Life. J. Gerontol. Nurs..

[70] Mousavi F., Tavabi A., Iran-Pour E., Tabatabaei R., Golestan B. (2012). Prevalence and Associated Factors of Insomnia Syndrome in the Elderly Residing in Kahrizak Nursing Home, Tehran, Iran. Iran. J. Public Health.

[71] Tsuzuki K., Sakoi T., Sakata Y. (2021). Effect of Seasonal Ambient Temperature on Sleep and Thermal Comfort in Older People Living in Public Elderly Facilities. Buildings.

[72] Rubiño J.A., Gamundí A., Akaarir M., Cañellas F., Rial R.V., Ballester N., Nicolau M.C. (2017). Effects of differences in the availability of light upon the circadian rhythms of institutionalized elderly. Chronobiol. Int..

[73] Shochat T., Martin J., Marler M., Ancoli-Israel S. (2000). Illumination levels in nursing home patients: Effects on sleep and activity rhythms. J. Sleep Res..

[74] Sloane P.D., Williams C.S., Mitchell C.M., Preisser J.S., Wood W., Barrick A.L., Hickman S.E., Gill K.S., Connell B.R., Edinger J. (2007). High-Intensity Environmental Light in Dementia: Effect on Sleep and Activity. J. Am. Geriatr. Soc..

[75] Kim D.E., Yoon J.Y. (2020). Factors that Influence Sleep among Residents in Long-Term Care Facilities. Int. J. Environ. Res. Public Health.

[76] Gentili A., Weiner D.K., Kuchibhatla M., Edinger J.D. (1997). Factors that disturb sleep in nursing home residents. Aging Clin. Exp. Res..

[77] Ellmers T., Arber S., Luff R., Eyers I., Young E. (2013). Factors affecting residents’ sleep in care homes. Nurs. Older People.

[78] Schnelle J.F., Ouslander J.G., Simmons S.F., Alessi C.A., Gravel M.D. (1993). The Nighttime Environment, Incontinence Care, and Sleep Disruption in Nursing Homes. J. Am. Geriatr. Soc..

[79] Schnelle J.F., Alessi C.A., Al-Samarrai N.R., Fricker R.D., Ouslander J.G. (1999). The nursing home at night: Effects of an intervention on noise, light, and sleep. J. Am. Geriatr. Soc..

[80] Giménez M.C., Geerdinck L.M., Versteylen M., Leffers P., Meekes G.J.B.M., Herremans H., de Ruyter B., Bikker J.W., Kuijpers P.M.J.C., Schlangen L.J.M. (2017). Patient room lighting influences on sleep, appraisal and mood in hospitalized people. J. Sleep Res..

[81] Martin D., Hurlbert A., Cousins D.A. (2018). Sleep Disturbance and the Change from White to Red Lighting at Night on Old Age Psychiatry Wards: A Quality Improvement Project. Arch. Psychiatr. Nurs..

[82] Grandner M.A. (2016). Sleep, Health, and Society. Sleep Med. Clin..

[83] UN Assembly (1948). Universal declaration of human rights. UN Gen. Assem..

[84] U.S. Congress (1949). Housing Act of 1949.

[85] Johnson A., Meckstroth A. (1998). Ancillary Services to Support Welfare to Work. https://aspe.hhs.gov/reports/ancillary-services-support-welfare-work.

[86] Cox R., Wenzel S., Rice E. (2016). Roadmap to a Unified Measure of Housing Insecurity. SSRN Electron. J..

[87] Virgile M.D.T., Katz J., Terry R., Graber J. (2019). Cognitive Pretesting of 2019 American Housing Survey Module on Housing Insecurity.

[88] Cornwell E.Y., Waite L.J. (2009). Measuring Social Isolation Among Older Adults Using Multiple Indicators From the NSHAP Study. J. Gerontol. Ser. B Psychol. Sci. Soc. Sci..

